# Supplementary material for: A Drastic Shift in Lipid Adducts in Colon Cancer Detected by MALDI-IMS Exposes Alterations in Specific K+ Channels
Source: Cancers (Basel). 2021 Mar 17;13(6):1350. doi: 10.3390/cancers13061350 (PMC8061771; doi:10.3390/cancers13061350)
Supplement: Supplementary file 1 [file cancers-13-01350-s001.pdf]

cancers

MDPI

Article

A Drastic Shift in Lipid Adducts in Colon Cancer Detected by MALDI-IMS Exposes Alterations in Specific K⁺ Channels

Jone Garate [1,†], Albert Maimó-Barceló [2,3,†], Joan Bestard-Escalas [2,3,‡], Roberto Fernández [1,4], Karim Pérez-Romero [2,3], Marco A. Martínez [2,5], Mª Antònia Payeras [2,6], Daniel H. Lopez [2,3], José Andrés Fernández [1] and Gwendolyn Barceló-Coblijn [2,3,*]

[1] Department of Physical Chemistry, University of the Basque Country (UPV/EHU), 48940 Leioa, Spain; jone.garate@gmail.com (J.G.); R.fernandez@imgpharma.com (R.F.); josea.fernandez@ehu.es (J.A.F.)
[2] Institut d'Investigació Sanitària Illes Balears (IdISBa, Health Research Institute of the Balearic Islands), 07120 Palma, Spain; albert.maimo@ssib.es (A.M.-B.); joanbe88@gmail.com (J.B.-E.); karim.perez@ssib.es (K.P.-R.); marco.martinez@ssib.es (M.A.M.); mariaantonia.payerascapo@ssib.es (Mª.A.P.); danielhoracio.lopezlopez@ssib.es (D.H.L.)
[3] Research Unit, Hospital Universitari Son Espases, 07120 Palma, Spain
[4] Research Department, IMG Pharma Biotech S.L., BIC Bizkaia (612), 48160 Derio, Spain
[5] Pathology Anatomy Unit, Hospital Universitari Son Espases, 07120 Palma, Spain
[6] Gastroenterology Unit, Hospital Universitari Son Espases, 07120 Palma, Spain
* Correspondence: gwendolyn.barcelo@ssib.es, Tel.: +34-871-205000 (ext. 66300)
† These authors contributed equally to this work.
‡ Current address: Université Catholique de Louvin, Brussels, Belgium; juan.bestard@uclouvain.be

Citation: Garate, J.; Maimó-Barceló, A.; Bestard-Escalas, J.; Fernández, R.; Pérez-Romero, K.; Martínez, M.A.; Payeras, Mª.A.; Lopez, D.H.; Fernández, J.A.; Barceló-Coblijn, G.; et al. A Drastic Shift in Lipid Adducts in Colon Cancer Detected by MALDI-IMS Exposes Alterations in Specific K⁺ Channels. *Cancers* **2021**, *13*, 1350. https://doi.org/10.3390/cancers13061350

Academic Editor: Isabelle Van Seuningen

Received: date
Accepted: date
Published: date

Publisher's Note: MDPI stays neutral with regard to jurisdictional claims in published maps and institutional affiliations.

Copyright: © 2021 by the authors. Submitted for possible open access publication under the terms and conditions of the Creative Commons Attribution (CC BY) license (http://creativecommons.org/licenses/by/4.0/).

Simple Summary: Colorectal cancer (CRC) is one of the most preventable yet deadliest cancers, one reason being that it involves very different lesions. Currently, there is a great international effort to improve CRC classification using as many molecular features as possible. A cutting-edge technique, imaging mass spectrometry, is used to enable the visualization of the bidimensional (2D) distribution of molecules across tissues in order to study how the composition of the cell membrane, in particular membrane lipids, changes in tumors. Our previous studies indicate that lipid composition is highly sensitive to cell alterations. Importantly, during the analysis, we are also able to establish changes in charged lipids, observations that can be misinterpreted. A close study of our results alongside information from public databases leads to the identification of gene coding for a potassium channel that could account for our observations and could represent a suitable target for drug development.

Abstract: Even though colorectal cancer (CRC) is one of the most preventable cancers, it is one of the deadliest, and recent data show that the incidence in people <50 years has unexpectedly increased. While new techniques for CRC molecular classification are emerging, no molecular feature is as yet firmly associated with prognosis. Imaging mass spectrometry (IMS) lipidomic analyses have demonstrated the specificity of the lipid fingerprint in differentiating pathological from healthy tissues. During IMS lipidomic analysis, the formation of ionic adducts is common. Of particular interest is the $[Na^+]/[K^+]$ adduct ratio, which already functions as a biomarker for homeostatic alterations. Herein, we show a drastic shift of the $[Na^+]/[K^+]$ adduct ratio in adenomatous colon mucosa compared to healthy mucosa, suggesting a robust increase in K^+ levels. Interrogating public databases, a strong association was found between poor diagnosis and voltage-gated potassium channel subunit beta-2 (KCNAB2) overexpression. We found this overexpression in three CRC molecular subtypes defined by the CRC Subtyping Consortium, making KCNAB2 an interesting pharmacological target. Consistently, its pharmacological inhibition resulted in a dramatic halt in commercial CRC cell proliferation. Identification of potential pharmacologic targets using lipid adduct information emphasizes the great potential of IMS lipidomic techniques in the clinical field.

Keywords: colorectal cancer; lipidomics; ion adducts; potassium channels

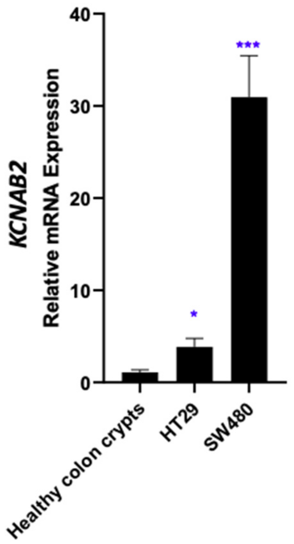

Figure S1. Gene expression levels of KCNAB2 in CRC commercial cell lines—Quantitative PCR. RNA was extracted from cultured cells using the TriPure™ Isolation Reagent (Sigma-Aldrich Chemie (Stenheim, Germany)) protocol. Briefly, samples were lysed in 1 volume of reagent, after which 1/3 of the volume in chloroform was added. Samples were set on ice for 10 min, and centrifuged at 12,000 ×g at 4 °C for 15 min. Following, the top layer was transferred to a new tube with 1 volume of isopropanol and left overnight at -20 °C. Samples were then centrifuged at 12,000 ×g at 4 °C for 10 min, and RNA was resuspended with DNA/RNAse-free water. Purity of RNA was assessed using the NanoDrop spectrophotometer (Nanodrop 2000, Thermo Fisher, Barcelona, Spain), considering a 260/280 ratio between 1.7-2.0 to be acceptable. Up to 1 µg of RNA was reverse-transcribe into cDNA using SensiFAST cDNA Synthesis Kit (Bioline, London, UK). Subsequent real-time PCR reactions were performed in CFX96 Real-Time System, C1000 Thermal Cycler (BIO-RAD) using Hard-Shell® 96-Well PCR Plates (Ref: #HSP9601, Bio-Rad, Barcelona, Spain), using Sensi Fast™ SYBR® No-Rox Kit (Bioline), PCR Water UltraPure 18.2MΩ, DNase/RNase-Free (Bioline). The following primers were used: 18S Fw 5′-TAAGCAACGA-GACTCTGGCAT-3′ and Rv 5′-CGGACATCTAAGGGCATCACAG-3′; KCNAB2 Fw 5′-CTGGAGTACGTGGATGTGGT-3′ and Rv:5′-CCACTTTCTCACGCTGGAAC-3′ (Isogen Live Science B.V., Utrecht, Netherlands). Gene expression levels of KCNAB2 in CRC commercial cell lines HT29 and SW480 relative gene expression to human healthy colon crypts of KCNAB2 (n = 34). Blue * represent unpaired t-test (* $p \leq 0.05$, *** $p \leq 0.0005$).

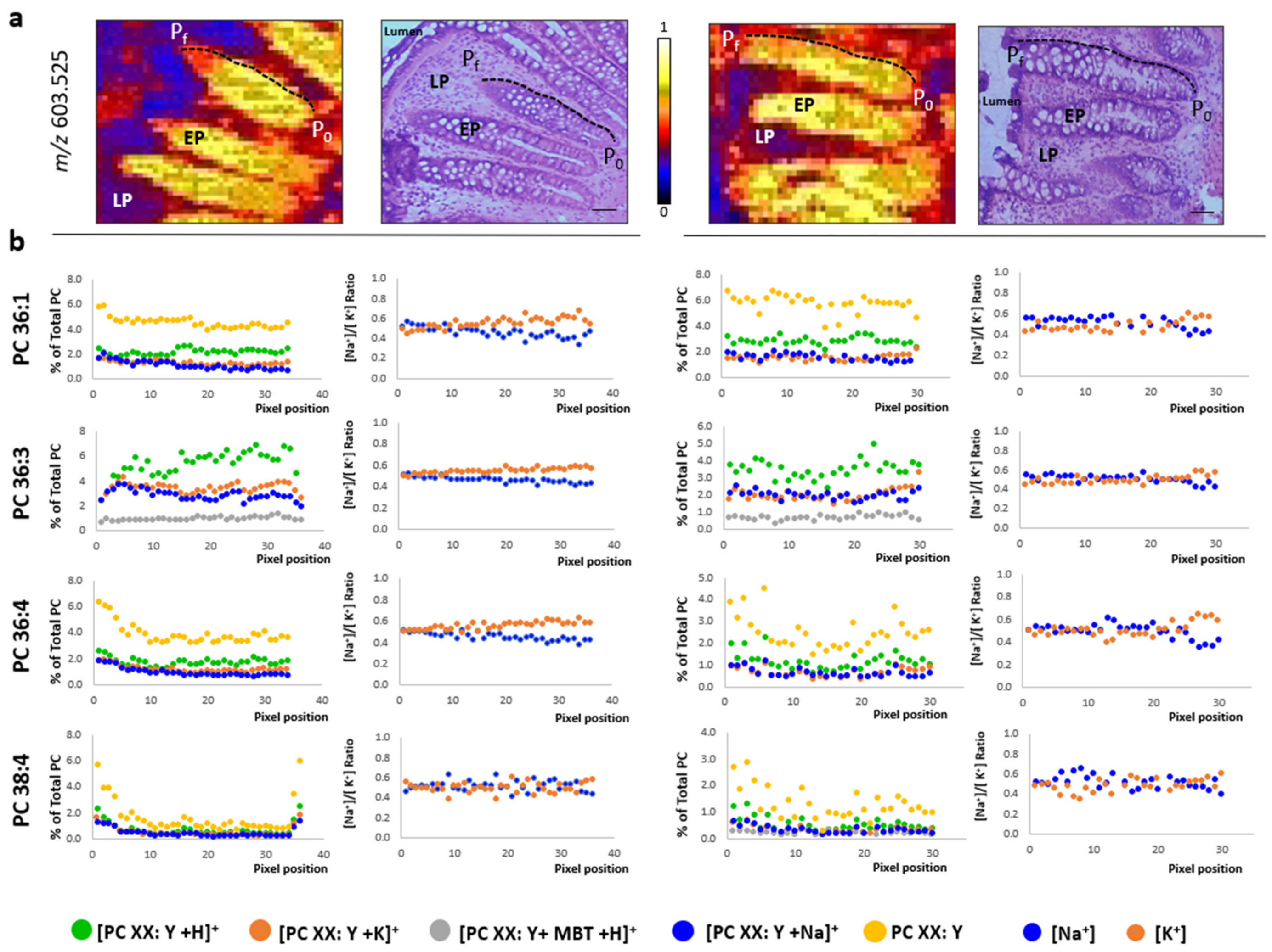

Figure S2. PC-adduct distribution in healthy colon epithelium. (**a**)—Selected MALDI-MS images obtained during the analysis in positive-ion mode (in particular m/z = 603.525) showing the paths depicted from the base (P_0) to the top (P_f) of the colon crypt to analyze, pixel by pixel, the changes in the lipidome along the healthy epithelium. Hematoxylin-eosin images of the consecutive section are included for comparison. Scale bar = 100 µm. (**b**)—Individual distribution of PC adducts along the depicted paths and the $[Na^+]/[K^+]$ ratio for selected PC species, PC 36:1, PC 36:3, PC 36:4, and PC 38:4. EP: epithelium; LP: lamina propria; P_0: first pixel of the path; P_f: final pixel of the path.

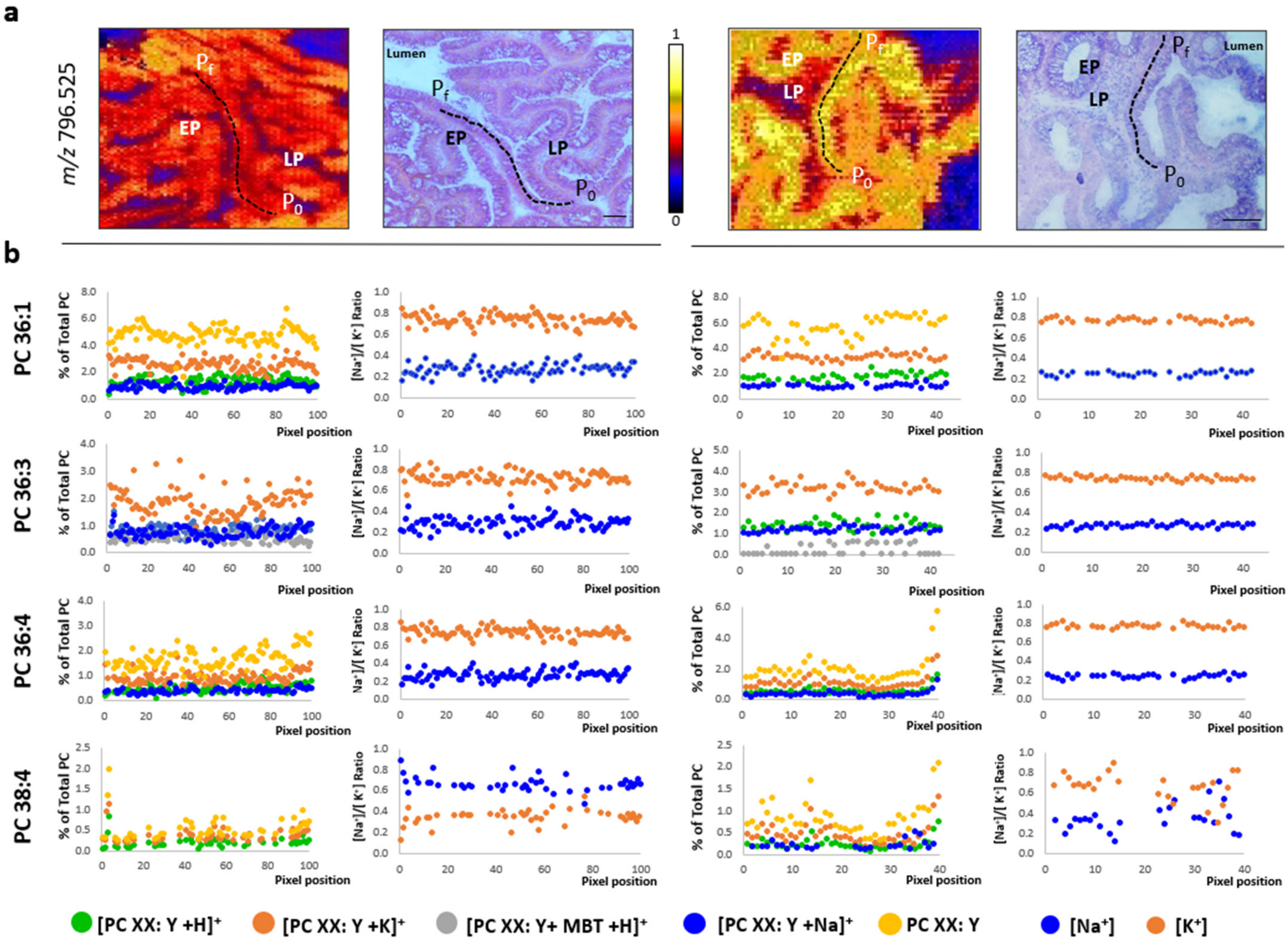

Figure S3. PC-adduct distribution in adenomatous colon epithelium. (**a**)—Selected MALDI-MS images obtained during the analysis in positive-ion mode (in particular m/z = 714.448) showing the paths depicted from the basal (P_0) to the luminal side (P_f) to analyze, pixel by pixel, the changes in the lipidome along the adenomatous epithelium. Hematoxylin-eosin images of the consecutive section are included for comparison. Scale bar = 100 µm. (**b**)—Individual distribution of PC adduct along the depicted paths and the $[Na^+]/[K^+]$ ratio for selected PC species, PC36:1, PC 36:3, PC 36:4, and PC 38:4. EP: epithelium; LP: lamina propria; P_0: first pixel of the path; P_f: final pixel of the path.

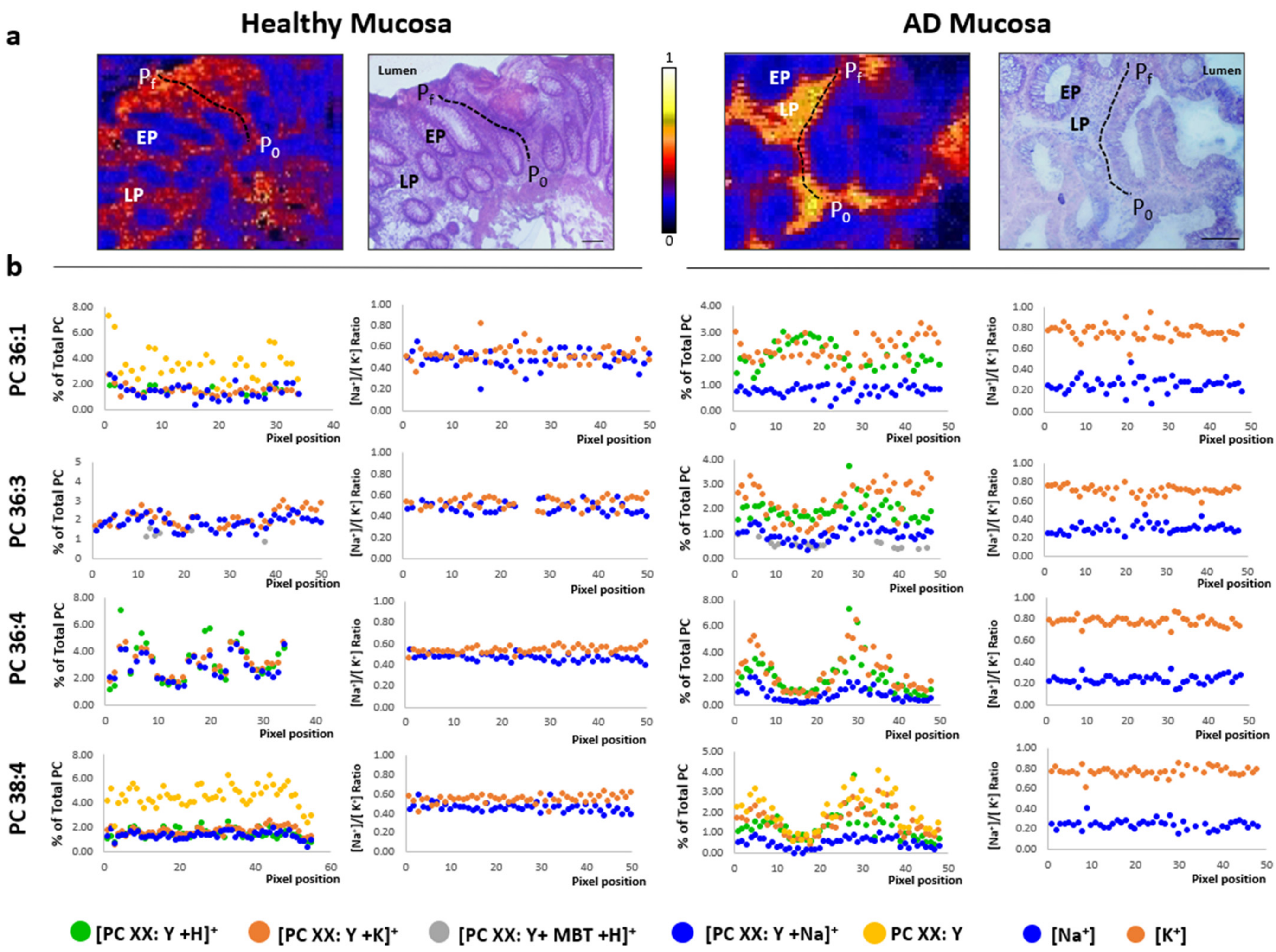

Figure S4. PC-adduct distribution in healthy colon and adenomatous polyp lamina propria. (**a**)—Selected MALDI-MS images obtained during the analysis in positive-ion mode (in particular m/z = 714.448) showing the paths depicted from the basal (P_0) to the luminal side (P_f) of the mucosa to analyze, pixel by pixel, the changes in the lipidome along the lamina propria in healthy and AD mucosa. Hematoxylin-eosin images of the consecutive section are included for comparison. Scale bar = 100 µm. (**b**)—Individual distribution of PC adduct along the depicted paths and the $[Na^+]/[K^+]$ ratio for selected PC species, PC 36:1, PC 36:3, PC 36:4, and PC 38:4. EP: epithelium; LP: lamina propria; P_0: first pixel of the path; P_f: final pixel of the path.

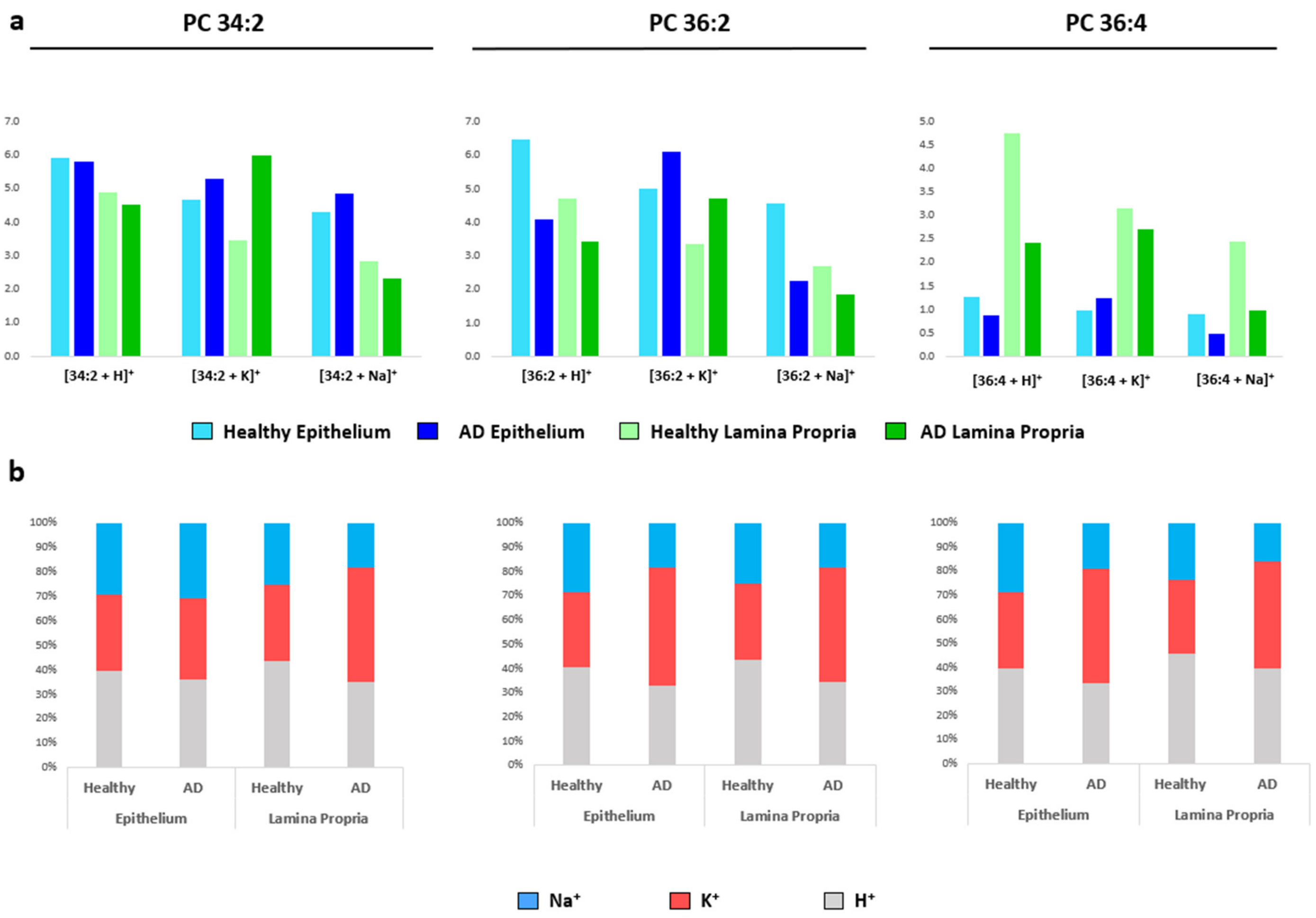

Figure S5. Homogenous impact of tumorigenesis on K^+ PC-adducts. (**a**)—Levels of lipid adducts of selected lipid species PC 34:2, PC 36:2, and PC 36:4 in healthy and AD mucosa. (**b**)—Normalized values showing that despite the impact of tissue malignization on the total levels of a particular lipid species, the level of the potassium adduct was always increased in the adenomatous counterpart.

a) Overall survival

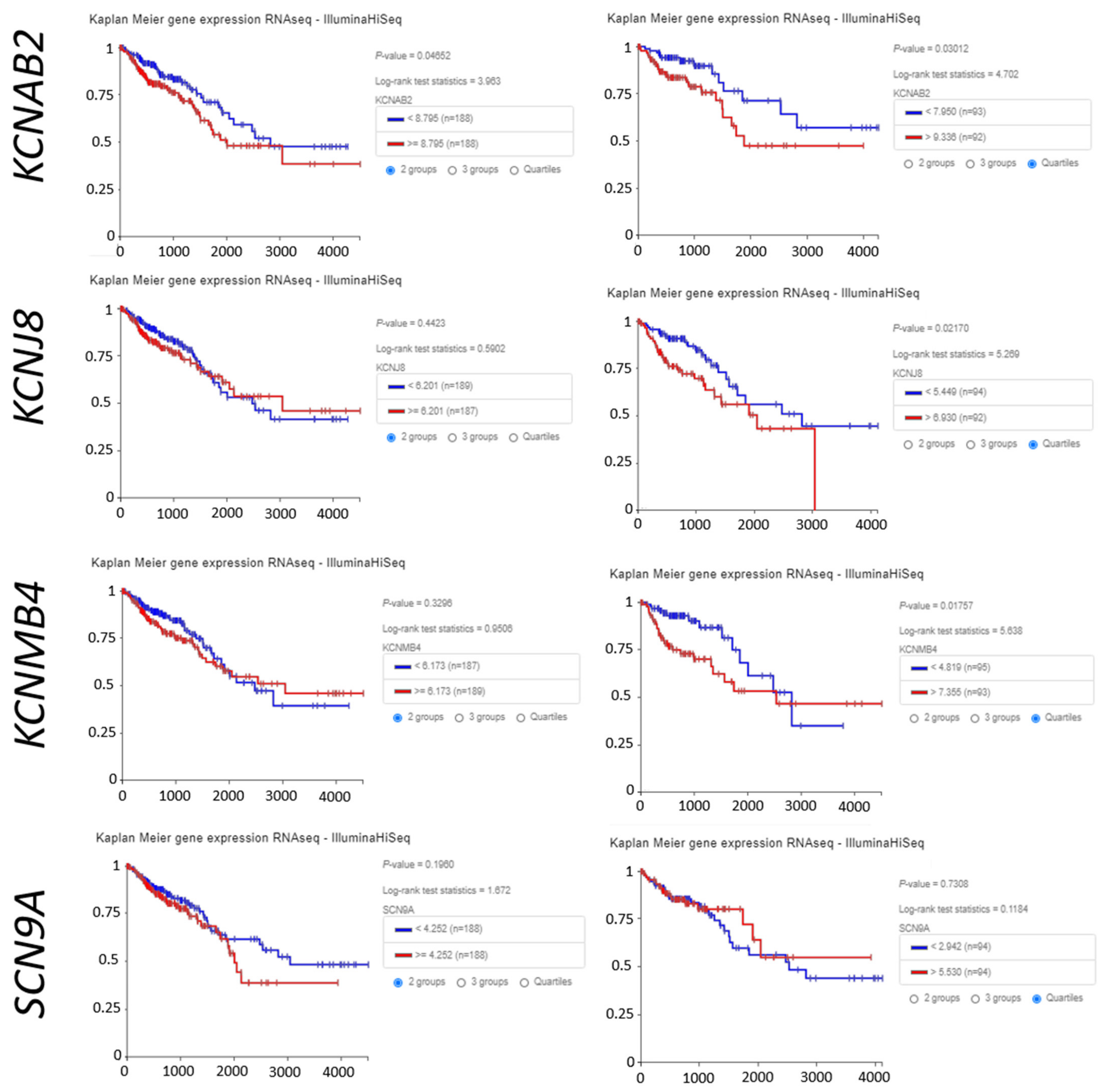

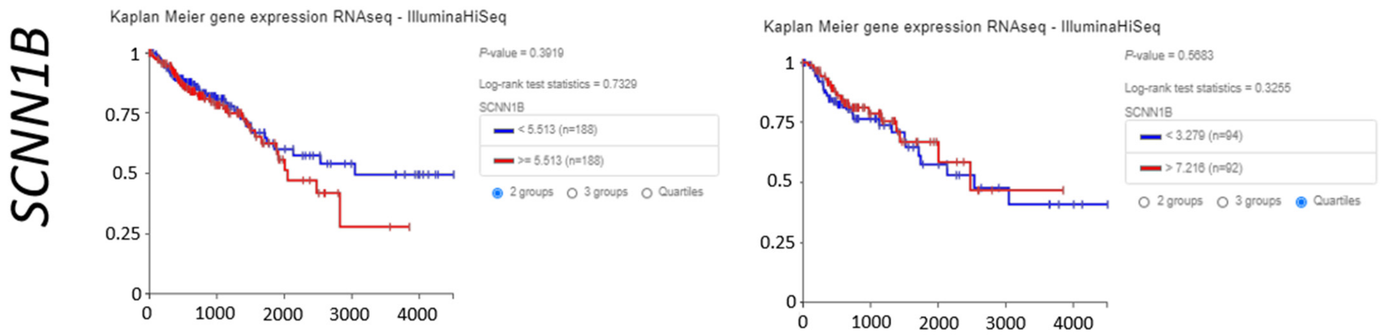

b) Disease specific survival

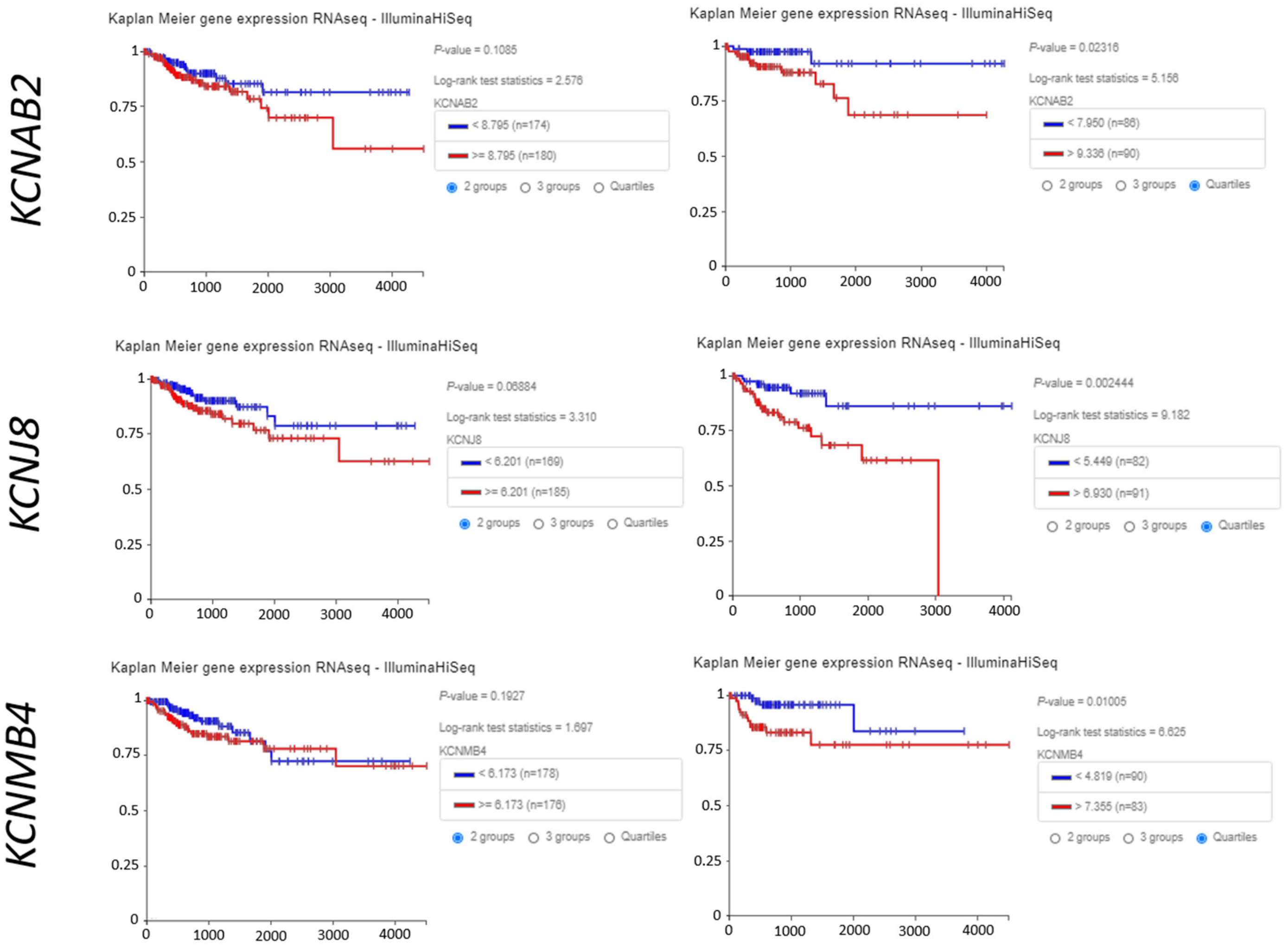

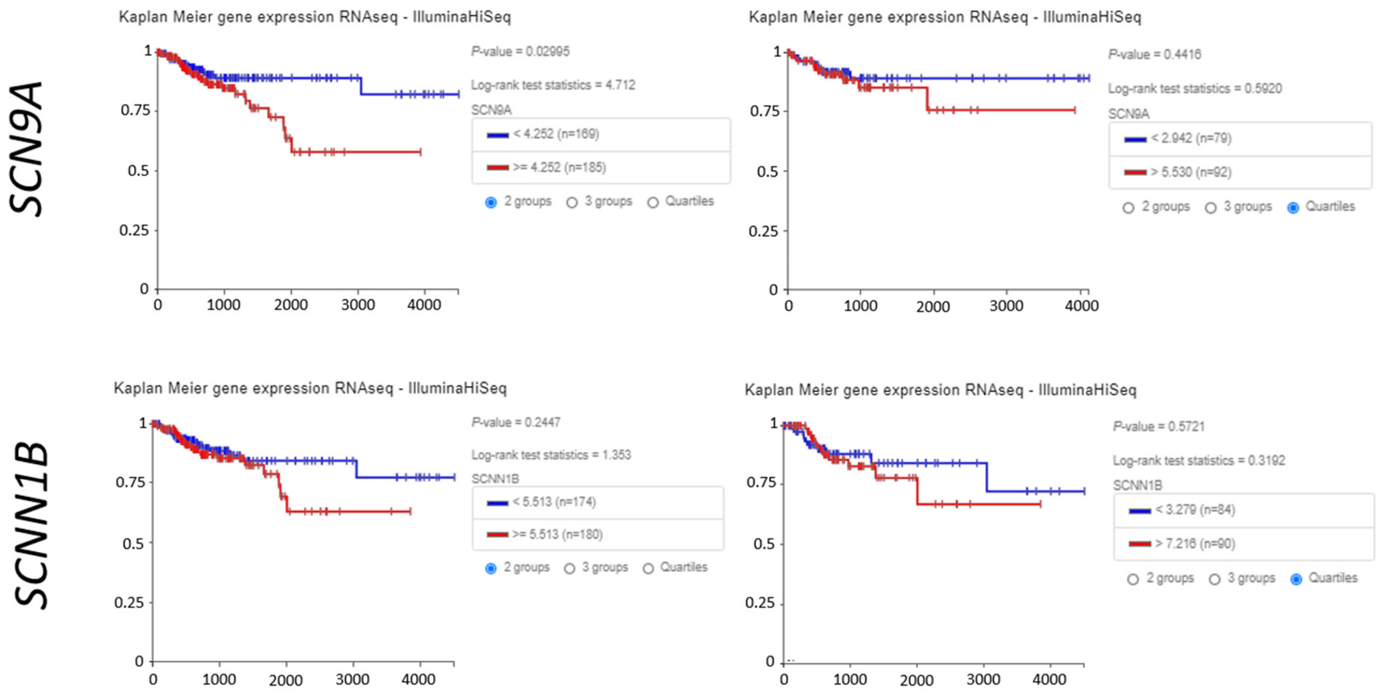

Figure S6. Kaplan-Meier survival analysis in CRC patients based upon *KCNJ8, KCNMB4* and *SCN1A* expression in primary colon tumors. Overall survival and disease specific survival rates based on UCSC Xena Browser Browser. Log-rank test (test statistics and *p*-value), for two groups (**a**) and quartile (**b**) RNAseq—Illumina HiSeq gene expression of primary tumor samples (*n*= 380 samples) from TCGA colon and rectal cancer (COADREAD) database.

Table S1. Log_2 fold change and associated *p*-value data for the comparisons made using GSE20916 and GSE35602-6480.

**GEO2R GSE20916 Distant Normal Colon Epithelium vs Carcinoma Epithelium**				
ID	Gene symbol	Log_2 FC	*p*-value	Adj. *p*-value
203402_at	*KCNAB2*	1.67	1.95E-02	3.94E-02
205303_at	*KCNJ8*	2.39	3.49E-06	3.19E-05
219287_at	*KCNMB4*	3.19	5.08E-07	7.24E-06
206950_at	*SCN9A*	-4.33	7.24E-12	1.82E-09
205464_at	*SCNN1B*	-2.58	6.13E-05	3.21E-04
GEO2R GSE20916 Normal colon vs Carcinoma				
ID	Gene symbol	Log_2 FC	*p*-value	Adj. *p*-value
203402_at	*KCNAB2*	0.31	0.0731	1.23E-01
205304_s_at	*KCNJ8*	1.19	0.0000436	1.84E-04
219287_at	*KCNMB4*	0.14	0.33	4.28E-01
206950_at	*SCN9A*	-0.71	0.00000123	7.91E-06
205464_at	*SCNN1B*	-4.35	4.03E-20	3.93E-17
GEO2R GSE35602-6480 Normal epithelium vs Tumor epithelium				
ID	Gene symbol	Log_2 FC	*p*-value	Adj. *p*-value
A_24_P151	*KCNAB2*	1.92	2.07E-03	2.13E-02
A_23_P64879	*KCNJ8*	0.97	0.107	2.25E-01
A_23_P64792	*KCNMB4*	1.41	0.131	2.56E-01
A_24_P3005	*SCN9A*	-3.86	1.06E-06	1.67E-04
A_32_P83098	*SCNN1B*	-2.28	6.11E-04	9.65E-03
GEO2R GSE35602-6480 Normal stroma vs Tumor stroma				
ID	Gene symbol	Log_2 FC	*p*-value	Adj. *p*-value
A_24_P151	*KCNAB2*	-0.86	1.75E-02	5.46E-02
A_23_P64879	*KCNJ8*	1.18	3.61E-02	8.76E-02
A_23_P64792	*KCNMB4*	-0.37	0.52800	6.37E-01
A_24_P3005	*SCN9A*	-1.66	1.96E-03	1.30E-02
A_32_P83098	*SCNN1B*	-1.11	3.80E-02	9.07E-02

Table S2. Fold change and associated *p*-value data for the comparisons made (CAFs vs CD133+, and FAP+ vs EPCAM+) using GSE34053 and GSE39396.

**GSE34053**				
HG-U133 Plus 2				
CAF vs CD133+				
**ID**	**Gene Symbol**	**FC**	***p*-value**	**FDR *p*-value**
222857_s_at	*KCNMB4*	-3.31	1.32E-05	0.0001
219287_at	*KCNMB4*	-2.2	0.0006	0.0033
234034_at	*KCNMB4*	-1.82	0.0003	0.002
203402_at	*KCNAB2*	-1.46	0.0005	0.0026
211791_s_at	*KCNAB2*	-1.45	0.0012	0.0056
205464_at	*SCNN1B*	-1.01	0.8805	0.929
205304_s_at	*KCNJ8*	1.71	6.34E-05	0.0005
205303_at	*KCNJ8*	2.3	5.93E-06	7.60E-05
229199_at	*SCN9A*	4.11	6.66E-09	3.75E-07
206950_at	*SCN9A*	4.27	1.10E-06	1.96E-05

**GSE39396**				
HT HG-U133 Plus PM				
FAP vs EPCAM				
**ID**	**Gene Symbol**	**FC**	***p*-value**	**FDR *p*-value**
203402_PM_at	*KCNAB2*	-1.1	0.2806	0.5565
211791_PM_s_at	*KCNAB2*	-1.03	0.951	0.9819
205303_PM_at	*KCNJ8*	1.54	0.0027	0.0276
205304_PM_s_at	*KCNJ8*	1.82	0.0027	0.0276
219287_PM_at	*KCNMB4*	-1.27	0.1881	0.4525
222857_PM_s_at	*KCNMB4*	-1.24	0.1218	0.3551
234034_PM_at	*KCNMB4*	-1.07	0.2243	0.4969
235930_PM_at	*KCNMB4*	-1	0.6289	0.8247
206950_PM_at	*SCN9A*	-1.03	0.6541	0.8395
229199_PM_at	*SCN9A*	-1.01	0.4971	0.7401
205464_PM_at	*SCNN1B*	-1.31	0.0297	0.1504
